# Supplementary figures and images for: RttA, a Zn2-Cys6 transcription factor in Aspergillus fumigatus, contributes to azole resistance
Source: Microbiol Spectr. 2025 Aug 21;13(10):e01810-25. doi: 10.1128/spectrum.01810-25 (PMC12502532; doi:10.1128/spectrum.01810-25)

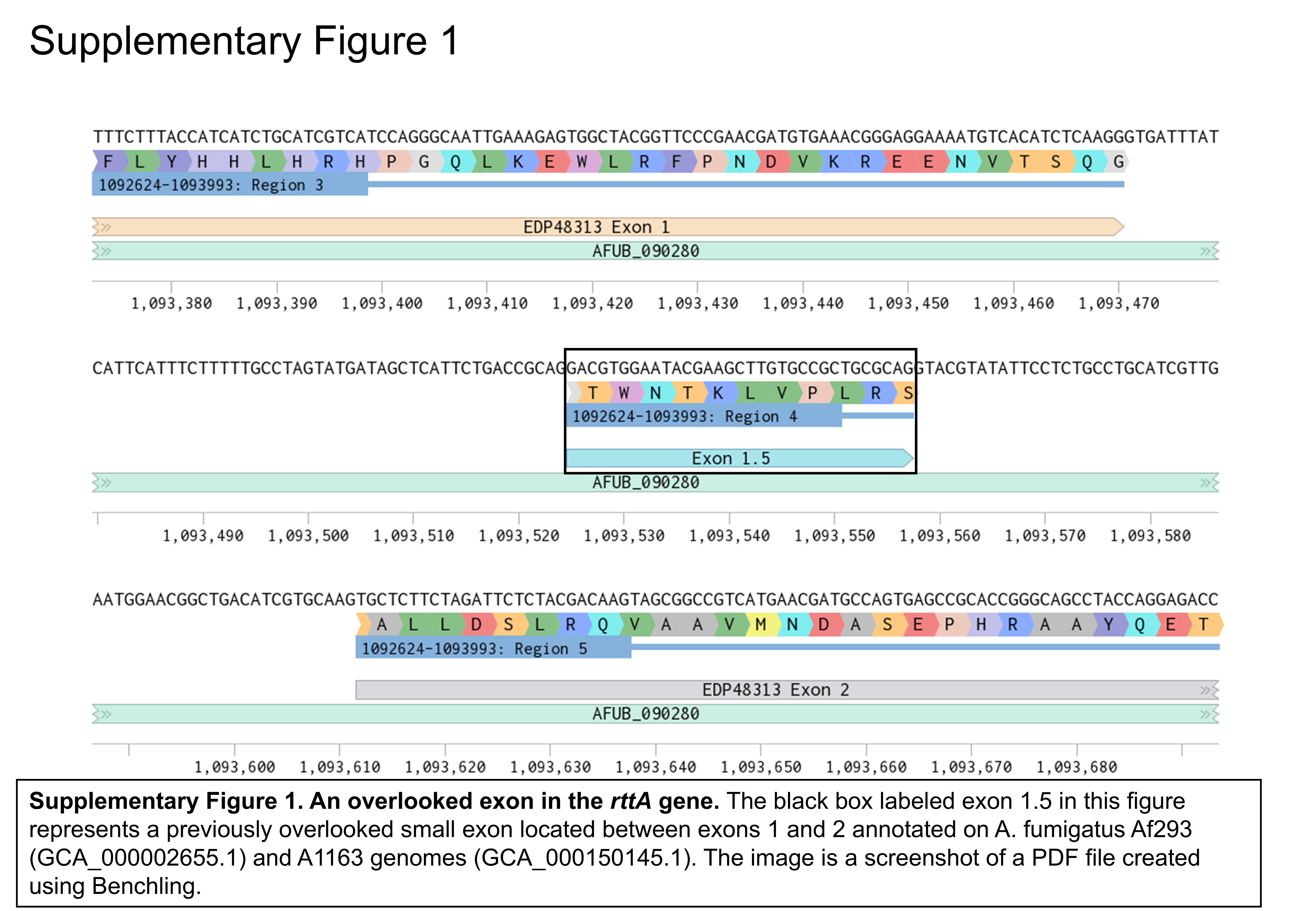

Supplement: Figure S1 — An overlooked exon in the rttA gene. [file spectrum.01810-25-s0001.tif]

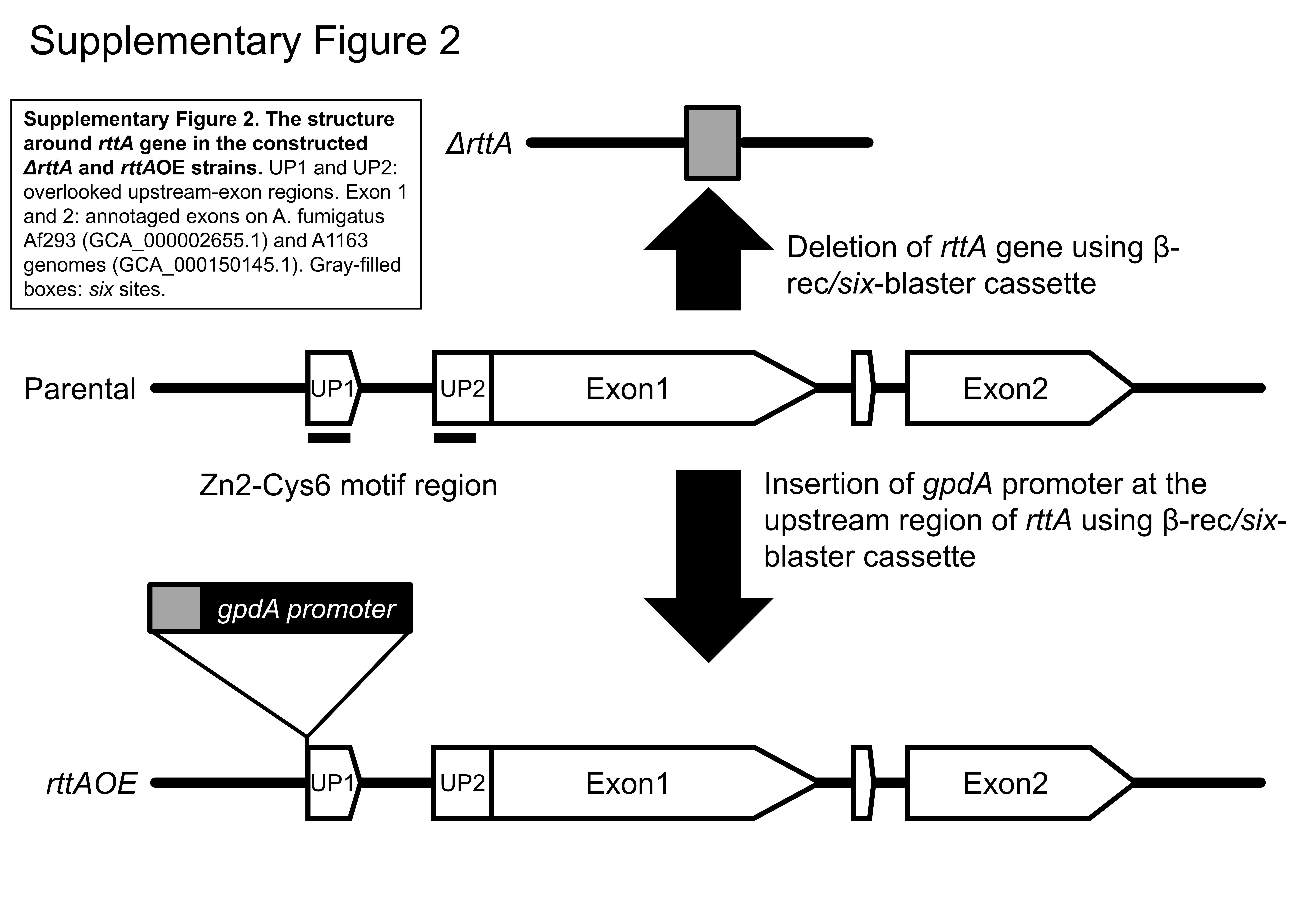

Supplement: Figure S2 — The structure around rttA gene in the constructed ΔrttA and rttAOE strains. [file spectrum.01810-25-s0002.tif]

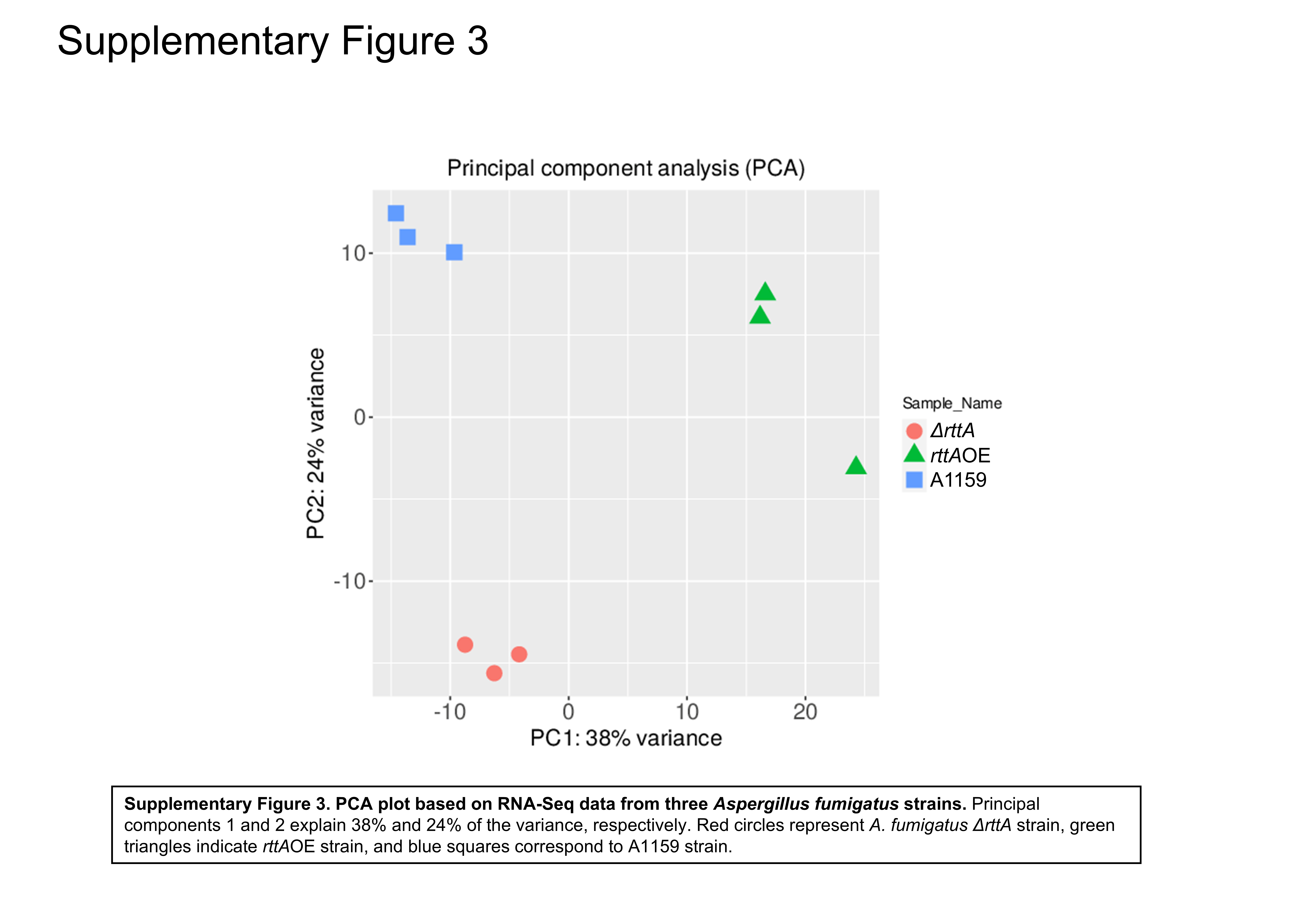

Supplement: Figure S3 — PCA plot based on RNA-Seq data from three Aspergillus fumigatus strains. [file spectrum.01810-25-s0003.tif]

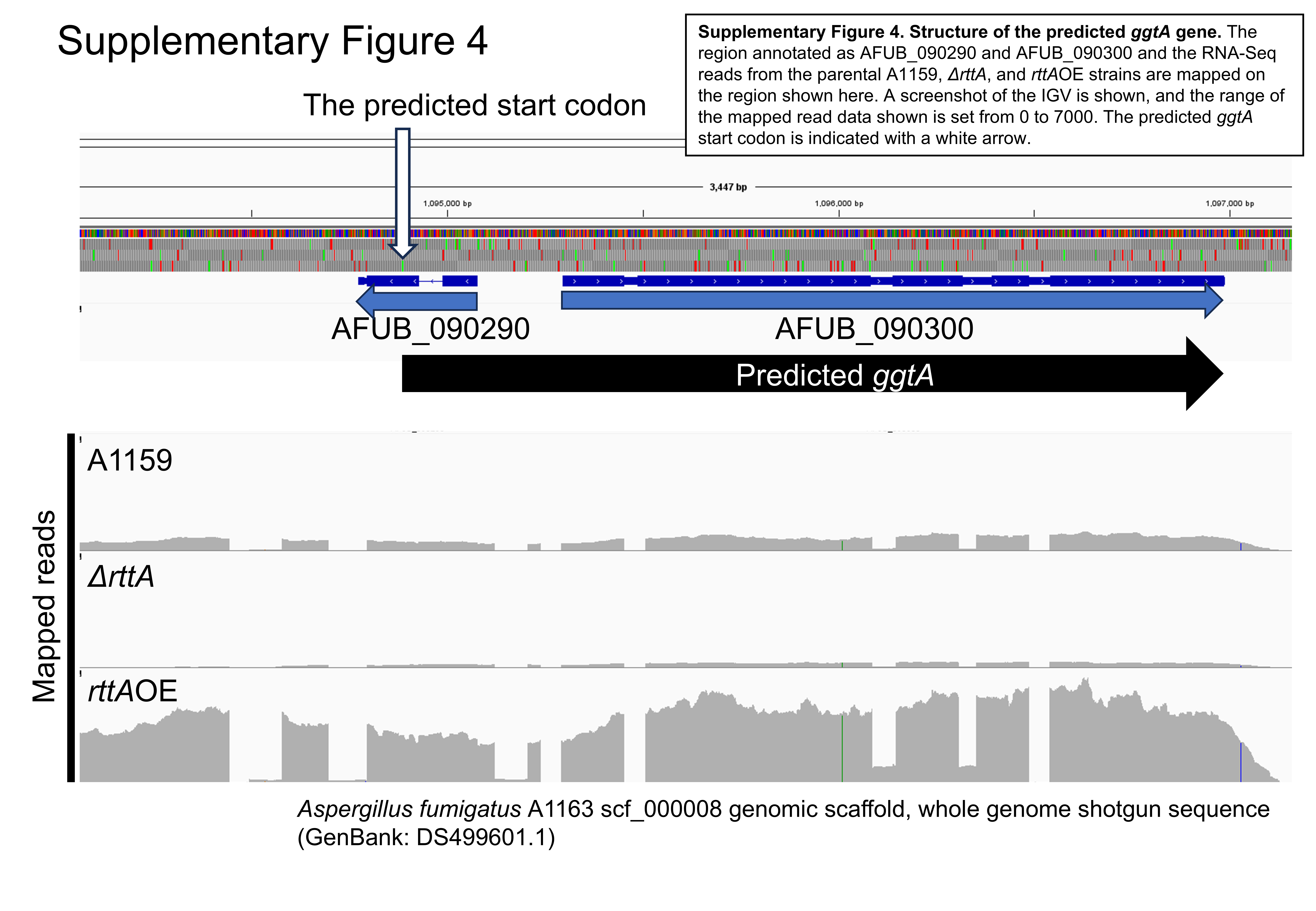

Supplement: Figure S4 — Structure of the predicted ggtA gene. [file spectrum.01810-25-s0004.tiff]

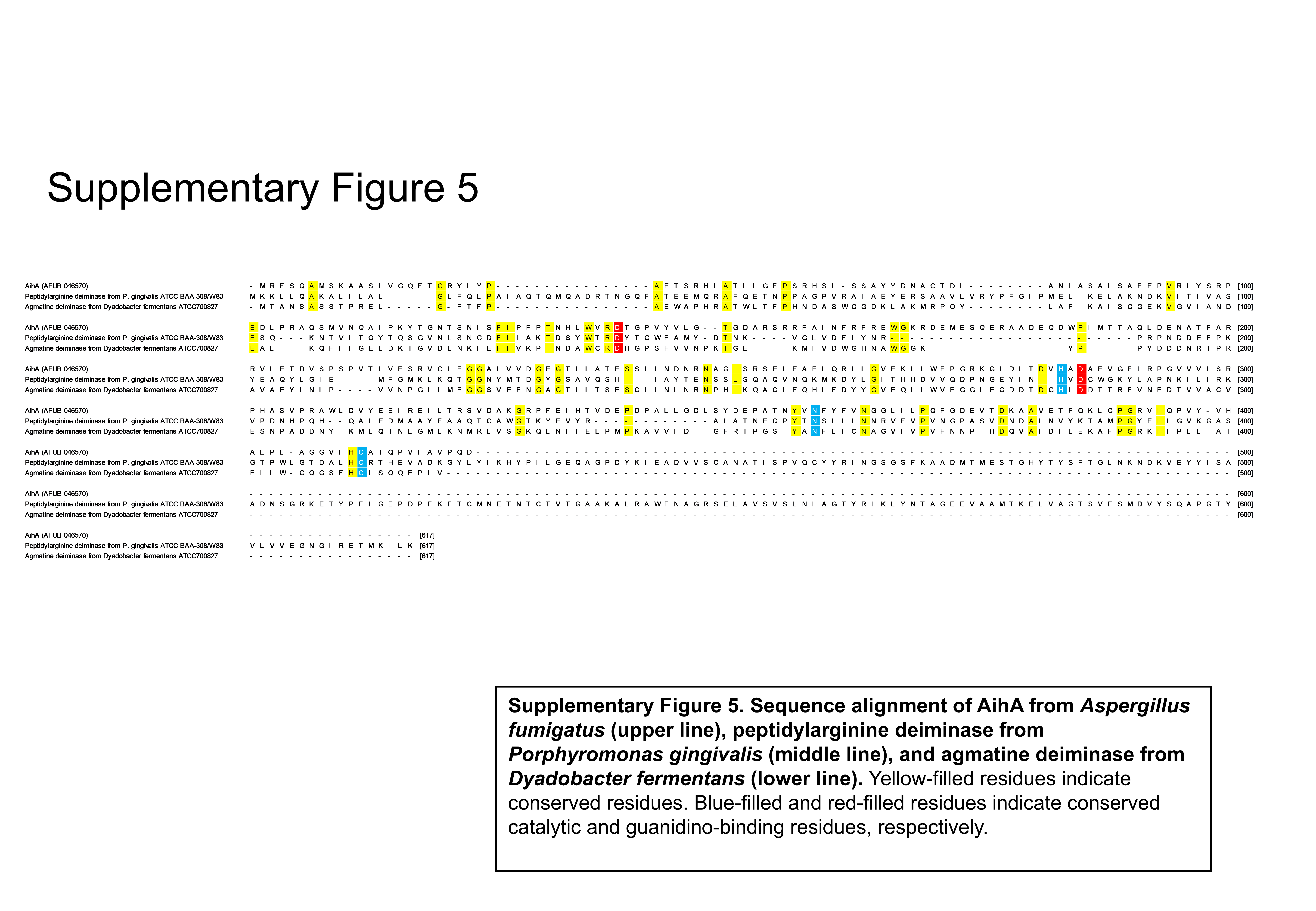

Supplement: Figure S5 — Sequence alignment of AihA from Aspergillus fumigatus (upper line), peptidylarginine deiminase from Porphyromonas gingivalis (middle line), and agmatine deiminase from Dyadobacter fermentans (lower line). [file spectrum.01810-25-s0005.tif]
